# Virtueller Praktikumstag Urologie: Studentenunterricht an der Technischen Universität München in Zeiten der COVID-19-Pandemie

**DOI:** 10.1007/s00120-020-01431-2

**Published:** 2021-01-12

**Authors:** Marc Kidess, Sebastian C. Schmid, Sebastian Pollak, Jürgen E. Gschwend, Pascal O. Berberat, Michael E. Autenrieth

**Affiliations:** 1 Klinik und Poliklinik für Urologie, Universitätsklinikum rechts der Isar, Technische Universität München, Ismaninger Straße 22, 81675 München, Deutschland; 2 TUM Medical Education Center, Fakultät für Medizin, Universitätsklinikum rechts der Isar, Technische Universität München, München, Deutschland

**Keywords:** Praktische Ausbildung, Video, Evaluation, Präsenzunterricht, Practical training, Video, Evaluation, Hands-on training

## Abstract

**Hintergrund und Einleitung:**

Die COVID-19-Pandemie stellte die Universitätskliniken vor eine große Herausforderung: Wie kann eine praktische Ausbildung angehender Mediziner ohne deren Präsenz in der Klinik erfolgen? Als Alternative zum regulären Praktikumstag haben wir innerhalb kurzer Zeit ein virtuelles Ersatzangebot mittels Lehrvideos zu unterschiedlichen urologischen Themen auf einer universitären Online-Plattform geschaffen. Ziel der Videos war die Vermittlung des Fachs Urologie in Theorie und Praxis.

**Material und Methoden:**

Die Videos wurden durch Mitarbeiter der Klinik anhand eines ausgearbeiteten Konzepts gefilmt und bearbeitet. Anschließend wurden diese Videos auf der universitären Online-Lehrplattform Moodle zur Verfügung gestellt. Zur Erfolgskontrolle mussten die Studierenden zu jedem Themenkomplex eine Frage beantworten. Eine Teilnahmebescheinigung wurde generiert, sobald die Studierenden mindestens 60 % aller Fragen richtig beantwortet hatten und an einer abschließenden Evaluation teilgenommen haben.

**Ergebnisse:**

Der virtuelle Praktikumstag wurde von 164 Teilnehmern absolviert. Die Evaluationen sind mit einem sehr positiven Gesamturteil ausgefallen – mit einer Schulnote von 1,2. Insgesamt war die Akzeptanz des Ersatzangebotes hoch.

**Diskussion:**

Das virtuelle Format als Alternative zum Präsenzunterricht wurde von den Studierenden sehr gut angenommen. Mit dem virtuellen Praktikumstag wurde eine schnelle und kontaktlose Alternative zum Präsenzunterricht geschaffen.

## Abstract (en)

**Background and introduction:**

The COVID-19 pandemic presents the challenge for medical education to teach practical skills without practical training. To provide an alternative to hands-on training during the COVID-19 lockdown, we created a virtual curriculum to teach practical skills using videos combined with online exams on a virtual e‑learning platform. The goal was to convey different theoretical and practical aspects of urology.

**Materials and methods:**

The videos were produced by department employees using a predefined concept. The students had access to the virtual curriculum via the university’s Moodle e‑learning platform. To assess the success of training, participating students had to pass an online exam about the curriculum’s contents, followed by an evaluation of the course.

**Results:**

A total of 164 participants took part in the virtual curriculum. The overall evaluation and feedback was very positive. The acceptance of the virtual alternative to hands-on teaching was high.

**Discussion:**

The virtual curriculum offered a fast and contactless alternative to the regular hands-on teaching.

## Hintergrund und Fragestellung

Die Lehre und Ausbildung angehender Mediziner stellt neben der Patientenversorgung und der Forschung den dritten Grundpfeiler einer Universitätsklinik dar. Praktischer Unterricht in Kleingruppen wie Blockpraktika und Praktikumstage sind dabei wichtige Instrumente für die praktische Ausbildung. Während Blockpraktika in der Regel einen Umfang von mehreren Wochen umfassen und somit eher den größeren Fächern wie Innere Medizin oder Chirurgie vorbehalten sind, dauern Praktikumstage meist ein bis zwei Tage.

Der Praktikumstag unserer urologischen Klinik richtet sich an die Studierenden im 9. Und 10. Semester nach erfolgreicher Teilnahme an der Hauptvorlesung Urologie. Der detaillierte Ablauf wurde 2014 von Schmid et al. näher vorgestellt [7]. Im Rahmen dieser Präsenzveranstaltung wurden verschiedene Seminare (Sexualanamnese, erektile Dysfunktion, digital-rektale Untersuchung) sowie eine OP-Hospitation und Hands-on-Training an Modellen (Zystoskopie, Kathetereinlage, Sonographie) für die Studierenden angeboten. Diese gut etablierte Lehrveranstaltung musste nun durch die COVID-19-Pandemie abrupt abgesagt werden.

Das generelle Verbot von Präsenzveranstaltungen stellt die studentische Ausbildung vor große Herausforderungen, da diese mit den Anforderungen der ärztlichen Approbationsordnung (ÄApprO; [1]) für den praktisch orientierter Unterricht vereint werden muss. Gleichzeitig gilt es, eine Verlängerung der Studienzeit möglichst zu vermeiden. Innerhalb kürzester Zeit mussten daher geeignete Alternativangebote zur herkömmlichen Präsenzlehre erstellt werden.

Deswegen wurde an unserer Klinik ein virtueller Praktikumstag entwickelt und erfolgreich durchgeführt, der den Studierenden einen strukturierten Einblick in das Fachgebiet und damit verbundene praktische Fertigkeiten geben sollte. Im Folgenden wird das Konzept und die Durchführung des digitalen Praktikumstages sowie die anschließende Evaluation vorgestellt. Durch die strukturierte Evaluation sollte die Zufriedenheit und der subjektive Lernerfolg der Studierenden im Rahmen des virtuellen Alternativangebotes untersucht werden.

## Material und Methoden

### Untersuchungskollektiv

Seitens der Fakultät waren 171 Studierende für die Teilnahme am Praktikumstag vorgesehen. Dabei handelte es sich überwiegend um Studierende aus dem 9. Und 10. Fachsemester, die bereits die Hauptvorlesung besucht und die Klausur im Fach Urologie bestanden hatten. Im Zeitraum vom 08.05.2020–07.08.2020 haben 164 Studierende den virtuellen Praktikumstag absolviert. Die Studierenden mussten zum erfolgreichen Absolvieren des Praktikumstages an einer Evaluation auf der Online-Plattform teilnehmen. Diese Evaluation bestand aus insgesamt 27 Fragen (3 Freitextfragen, 24 Multiple-choice-Fragen). Der Fokus wurde dabei auf die Studierendenzufriedenheit bezüglich des virtuellen Formats, der Didaktik, der Videogestaltung (Länge, Dozenten etc.) sowie der generellen Akzeptanz virtueller Angebote gelegt.

### Online-Plattform und verwendete Tools

Als Basis für den Online-Praktikumstag wurde die Plattform www.moodle.tum.de der Technischen Universität München verwendet. Auf dieser Plattform kann man in einem Baukastensystem unterschiedliche Lern- und Lehrelemente einfügen, z. B. Arbeitsblätter oder Videos. Die Videos wurden über Links eingebettet. Hinterlegt waren die Videos im mp4-Format im von der Universität genutzten System *mediasite*. Die Videos besaßen eine Größe von 36–1015 MB. Die Länge der durch uns produzierten Videos lag zwischen 2:02 und 25:17 min.

Die Videos wurden mithilfe der Systemkamera *Sony α 6600* gedreht und mit dem Programm „*Windows Movie Maker“* (Version 2012) bearbeitet.

Um sich in den Online-Kurs einschreiben zu können, erhielten die Studierenden einen einheitlichen Einschreibeschlüssel. Von der Idee bis zur Fertigstellung des virtuellen Praktikumstages vergingen drei Wochen. Am 08.05.2020 wurden die Studierenden per E‑Mail über den Online-Praktikumstag informiert. Dieser war zu diesem Zeitpunkt bereits freigeschaltet. Die Studierenden konnten ihn bis zum offiziellen Ende der Vorlesungszeit (07.08.2020) absolvieren.

### Entwicklung und didaktische Struktur der Lehrvideos

Unser Konzept sah die Erstellung von Lehrvideos zu unterschiedlichen Themen vor, das den Studenten das praktische und klinisch-relevante Alltagswissen aus der Urologie näherbringen sollte.

Die Videos wurden nach Entwicklung eines detaillierten Drehbuchs für das jeweilige Video mit den Dozenten gefilmt. Der grundsätzliche Aufbau war wie folgt:

- Begrüßung und Einführung ins Thema (ca. 1–2 min),
- Darstellung des Themas (ca. 5–15 min),
- Zusammenfassung im Hinblick auf die alltagsrelevanten Fakten, die jeder Arzt wissen sollte (ca. 1–2 min).

Manche der Aufnahmen erfolgten unter Mitwirkung von Patienten oder PJ-Studierenden, meist wurden die Videos aber mithilfe von Modellen (z. B. Zystoskopiephantom) oder als Frontalvortrag gestaltet. Das Filmen und die Bearbeitung der Lehrvideos wurde von zwei sehr engagierten studentischen Hilfskräften innerhalb von 3 Wochen realisiert. Die Videos kann man grob in vier Kategorien einordnen: *Seminarvideos, Videos zur Vermittlung praktischer Fertigkeiten, OP-Videos* und *Andere* (z. B. Rundgang durch die Klinik). Die Themen der Lehrvideos sind in Tab. 1 dargestellt.

**Table 1:** 

Thema Video	Inhalt	Benutzte Hilfsmittel
Einführung (2:02 min)	Einführung und Erläuterung des Konzepts	–
Die Klinik und Poliklinik für Urologie in Zeiten der COVID-19-Pandemie (6:25 min)	Führung durch die Poliklinik mit Schwerpunkt auf die Corona-bedingten veränderten Abläufe, z. B. im Rahmen der onkologischen Versorgung	–
Prästationäres Management (2:11 min)	Einführung in den Ablauf der OP-Vorbereitungen	–
OP-Clip: endourologische OP – Holmiumlaserenukleation der Prostata (17:55 min)	Demonstration einer lasergestützten Adenomenukleation mit Erklärung und Präsentation des Instrumentariums	–
OP-Clip: minimal-invasive, roboterassistierte Blasenteilresektion bei Urachuszyste (14:58 min)	Demonstration von Robotersystem und Konsole mit intraoperativen Videosequenzen	Foto der resezierten Urachuszyste
DRU (13:19 min)	Strukturiertes Vorgehen bei der DRU und der Untersuchung der Prostata mit Erläuterung der Pathologien	Verwendungen von anatomischen Abbildungen, Abbildungen zu unterschiedlichen Pathologien (z. B. Analprolaps) und Benutzung einer Flipchart zur didaktischen Aufbereitung der Thematik; Veranschaulichung der Untersuchungstechnik am Modell
Urologische Sonographie (16:51 min)	Erläuterung des Ultraschallgeräts sowie, des Vorgehens bei einer strukturierten Untersuchung. Schrittweise Demonstration der sonographischen Untersuchung von Niere, Harnblase und Prostata (transabdominal)	Veranschaulichung der Untersuchungstechniken (inklusive Restharnbestimmung) mit Hilfe eines PJ-Studenten, Klinikinformationsplakat, Untersuchungsbefund eines rektalen Ultraschalls der Prostata mit Prostatakarzinom
Uroflowmetrie (6:25 min)	Erläuterung der Indikationen sowie des Funktionsmechanismus der Untersuchung. Praktische Besprechung eines Befunds und Präsentation von pathologischen Befunden, Präsentation der Geräte	Untersuchungsbefund, Lehrbuchabbildung
Zystoskopie (10:54 min)	Demonstration der Zystoskopie am Modell mit Erklärung zu starrem und flexiblem Zystoskop mit Zusammenbau und Klärung der Indikationen	Demonstration am Zystoskopiemodell, Präsentation von möglichen Zystoskopiebefunden anhand von Modellen sowie von starrem und flexiblem Zystoskop
Katheterlehre (11:13 min)	Demonstration der verschiedenen Katheterarten (DJ etc.) mit Fokus auf die wichtigen Unterschiede und praktische Anwendung	Vorführung der unterschiedlichen Katheter, Verwendung der Flipchart
Einlage eines transurethralen Dauerkatheters (10:42 min)	Vorbereitung und Durchführung am Modell und am Patienten mit Fokus auf Fallstricke und Komplikationen	Demonstration am Modell und am Patienten
Anlage eines suprapubischen Blasenkatheters (4:09 min)	Präsentation der notwendigen Utensilien und erklärte Durchführung am Patienten	Demonstration am Patienten
Harnwegsinfektionen (16:10 min)	Systematische Präsentation der Harnwegsinfektionen mit anschließender praktischer Demonstration der Diagnostik	Flipchart, Demonstration des Urinschnelltests
Hodenkarzinom (13:51 min)	Vortrag zum Hodenkarzinom mit besonderem Fokus auf Klinik, Diagnostik, Früherkennung und Therapie	–
Prostatakarzinom (10:30 min)	Vortrag zum Prostatakarzinom mit besonderem Fokus auf die PSA-Untersuchung, Diagnostik, Therapie, Altersverteilung und Prognose mit Veranschaulichung anhand von anatomischen Abbildungen	Anatomische Abbildungen
Komplikationsmanagement nach radikaler Prostatektomie (12:14 min)	Einführung in die operativen Möglichkeiten bei Inkontinenz und erektiler Dysfunktion	Videobeispiele zur erektilen Dysfunktion und Inkontinenz, Präsentation der Implantate
Harnblasenkarzinom (6:19 min)	Vermittlung der pathophysiologischen, klinischen, diagnostischen und therapeutischen Grundlagen	Anfertigung von Zeichnungen auf einer Flipchart
Medikamentöse Tumortherapie (8:48 min)	Überblick über die Möglichkeiten der medikamentösen Tumortherapie bei Prostata‑, Blasen- und Hodenkarzinom	–
Andrologie (25:17 min)	Überblick über die diagnostischen und therapeutischen Möglichkeiten bei erektiler Dysfunktion	Verwendung von Modellen und Devices (z. B. Unterdruckpumpe)

*DRU* digital-rektale Untersuchung, *PSA* prostataspezifisches Antigen, *PJ* praktisches Jahr, *OP* Operation

**Figure 1:**
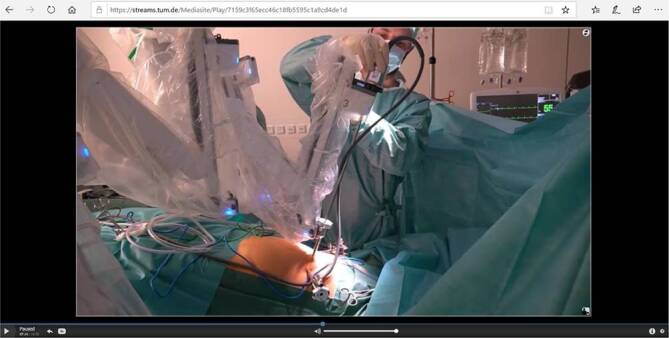

**Figure 2:**
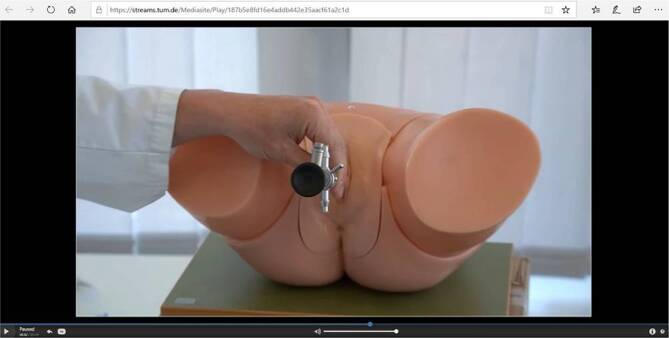

In den Videos wurden verschiedene Lehrmittel verwendet, um dem Film einen übersichtlichen didaktischen Aufbau zu geben (z. B. Flipcharts mit Mindmaps, anatomische Abbildungen etc.). Des Weiteren wurden Links zu anderen Videos eingefügt (Abb. 1) (z. B. Image- oder Operationsfilmen der Fachgesellschaft). In den Videos zum Erlernen praktischer Fertigkeiten (z. B. Einlegen eines transurethralen Dauerkatheters) wurde ein besonderer Fokus auf die einzelnen Handlungsschritte und das Erkennen von Fallstricken gelegt (Abb. 2).

Insgesamt waren 16 Dozierende und 2 studentische Hilfskräfte an der Umsetzung des virtuellen Praktikumstages beteiligt.

### Anwesenheits- und Lernerfolgskontrolle

Um eine Leistungs- und Anwesenheitskontrolle zu gewährleisten wurde zu jedem Thema eine Multiple-choice- (MC-)Frage gestellt (insgesamt 12 Fragen). Für die Beantwortung hatte der Studierende einmalig 3 min Zeit. Jede Frage wurde von den Dozenten gemeinsam mit den studentischen Hilfskräften entworfen. Der Inhalt der Frage sowie die dazugehörigen richtigen und falschen Aussagen bezogen sich auf das Video. Es wurde bei der Erstellung darauf geachtet, dass die Fragen praktisch relevante Wissensinhalte abfragen, die ein Arzt jeder Fachrichtung beherrschen sollte.

Die von uns verwendete Plattform bietet die Möglichkeit, ein Zertifikat als Teilnahmebescheinigung zu generieren. Um die Teilnahmebescheinigung herunterladen zu können, mussten folgende Bedingungen erfüllt sein: Jede Testfrage musste beantwortet werden und über alle Testfragen musste ein korrektes Ergebnis von mindestens 60 % erreicht werden. Bei Unterschreiten dieser Grenze war die Möglichkeit eines mündlichen Nachtestats vorhanden.

**Figure 3:**
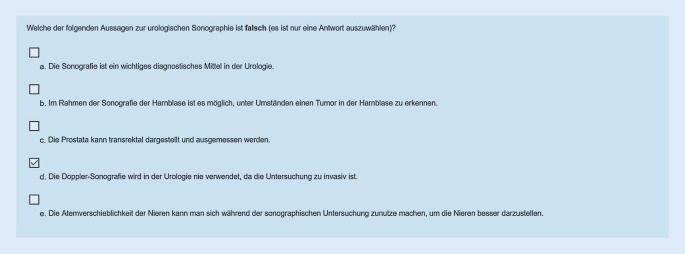

Zudem mussten die Studierenden im Anschluss an die Lehrvideos eine Online-Evaluation ausfüllen. Nach abgeschlossener Evaluation erhielten die Studierenden in einem Abschlusstext ein Kennwort, das sie zum Erhalt des Zertifikats eingeben mussten. Das automatisch generierte Zertifikat konnten die Studenten anschließend auf der Webseite der Fakultät/des Dekanats (www.meditum.de) hochladen und so, als Alternative zum üblichen Laufzettel, ihre Online-Anwesenheit nachweisen. (Abb. 3).

## Fragen und Ergebnisse der Evaluation

**Figure 4:**
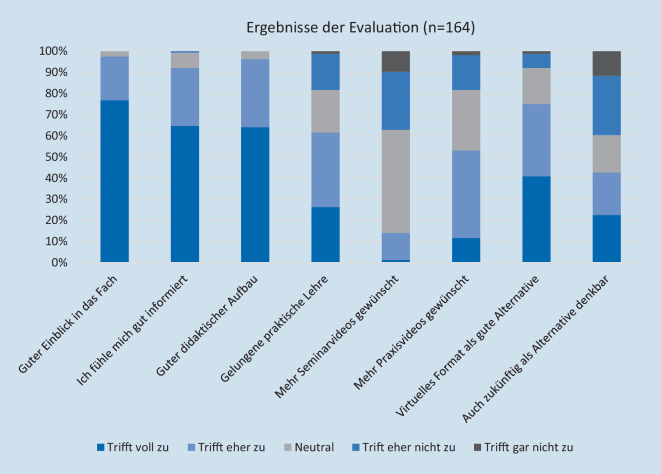

Ein Teil der Ergebnisse der Evaluation werden in Abb. 4 dargestellt. Zu der Evaluation ist anzumerken, dass alle Kursteilnehmer an der Evaluation teilgenommen haben. Hierbei handelt es sich um ein erstes Meinungsbild eines Semesters, welches durch weitere Evaluationen zukünftiger Kollektive/Semester von Kursteilnehmern bestätigt werden muss.

Auf die Frage, für wie wahrscheinlich es die Teilnehmer halten, dass sie nach Absolvierung des Praktikumstages einen Facharzt für Urologie anstreben, antworteten 35,8 %, dass sie es zumindest in Betracht ziehen. 23,8 % der Studierenden gaben an, dass sie bezüglich der Facharztwahl noch unentschlossen sind. 1,2 % wollten bereits vor Absolvierung des Praktikumstages Urologe werden.

Als Schulnote erhielt der Kurs eine Durchschnittsnote von 1,2 (Note 1: 81,1 %; Note 2: 17,7 %; Note 3: 1,2 %).

In den Freitextantworten erwähnten die Studierenden häufig, dass, sie trotz Wertschätzung des virtuellen Angebotes dieses Format nicht als ebenbürtig zur Präsenzveranstaltung sehen, da die Vermittlung praktischer Tätigkeiten nur durch selbständiges Üben gelingen kann. Manche Studierende hätten sich mehr Informationen zum Berufsbild gewünscht (z. B. Arbeitszeiten, Gehalt etc.). Zahlreiche Studierende gaben an, „Spaß beim Lernen“ und „Lust auf mehr“ gehabt zu haben. Auch wurde mehrfach angeraten, die Videos in Zukunft additiv zum Präsenzpraktikumstag oder in anderer Form anzubieten. Ein paar Zitate:

„Ich war erst enttäuscht über den Ausfall, muss aber sagen, dass mir besonders gefallen hat, die praktischen Tätigkeiten so nah und genau zu sehen. Ich weiß nicht, ob das vor Ort mit vielen Studenten in der Form möglich gewesen wäre.“

„Der PT Urologie war das einzige wirklich sehr gute Ersatzangebot. […] Die Videos waren wirklich super, die Fragen waren nicht zu schwer und alles in allem eine Klasse Alternative. Dass gerade aus der Urologie, als eher kleinerem und unscheinbaren Fach, die super Online-Lehre kommt, hätte ich nicht gedacht. […] Sehr sehr gut!“

## Ergebnisse der Anwesenheits- und Lernerfolgskontrolle

Alle 164 Teilnehmer bestanden die Anwesenheits- und Lernerfolgskontrolle (Range 66,67–100 %).

## Diskussion

Aufgrund der COVID-19-Pandemie hat sich in kürzester Zeit das Umfeld der universitären Lehre in der Medizin stark verändert, wie auch diverse neuere Publikationen zeigen [3, 6, 8–10]. Um diesen neuen Umständen und den Zwängen des Infektionsschutzes gerecht zu werden, hat die Klinik für Urologie der Technischen Universität München einen digitalen Praktikumstag aufgebaut. Dieser Praktikumstag sollte in kurzer Zeit zur Verfügung stehen, dabei praktisch orientiert sein und den Studierenden möglichst umfassenden Einblick in das Fach liefern.

Es wurde innerhalb weniger Wochen eine virtuelle Möglichkeit geschaffen, einen praktisch orientierten Kurs zum Erwerb der ärztlich relevanten Tätigkeiten anzubieten, wodurch die Anforderungen der ÄApprO modifiziert und angepasst an die COVID-19-Situation erfüllt wurden. Durch Videos zu verschiedenen Themen konnte zudem ein umfangreicheres propädeutisches Angebot als im Präsenzpraktikumstag möglich gemacht werden, und damit die Qualität der theoretischen Lehrinhalte gesteigert werden. Die Themen wurden von Dozierenden mit Spezialisierung auf das jeweilige Thema vorgetragen. Auch wenn die Vermittlung praktischer Fertigkeiten ohne Präsenz nur begrenzt möglich ist, konnte dies insgesamt gut vermittelt werden, was auch die Evaluation entsprechend widerspiegelt. Die Kursteilnehmer gaben ein sehr positives Gesamturteil (Schulnote 1,2) ab; insbesondere die Freitextantworten spiegelten dies eindrücklich wider. Das Feedback der Teilnehmer zeigt aber auch, dass eine Präsenzveranstaltung mit praktischem Üben weiterhin gewünscht wird; ein virtueller Ersatz der Praxis ist letztendlich nicht möglich. Dennoch können sich 42,7 % sogar vorstellen diese virtuelle Praktikumstagversion auch zukünftig als Alternative zum regulären praktischen Unterricht zu nutzen. In den Freitextantworten wurde wiederholt angemerkt, das virtuelle Angebot künftig als Erweiterung zum regulären Präsenzunterricht anzubieten.

In der Fachliteratur sind ähnliche Erfahrungen einer virtuellen praktischen Lehre beschrieben. Shih et al. beispielsweise haben, neben Online-Videos zur Beibringung praktischer Tätigkeiten, auch noch Vorbereitungsblätter und eine anschließende einstündige virtuelle Livekonferenz in Kleingruppen abgehalten. Trotz positiver Erfahrungen kommt man sowohl hier, als auch in anderen Fachartikel zum Schluss, dass die virtuelle Lehre die Präsenzveranstaltungen mit praktischem Üben am Patienten nicht ersetzen kann [8, 9]. Auch in vorklinischen Fächern, wie z. B. in der praktischen anatomischen Lehre, wurde auf virtuelle Ersatzangebote wie Videos oder virtuelle Konferenzen zurückgegriffen, mit überwiegend ebenso guten Rückmeldungen [3, 9].

Weiterhin anzumerken ist, dass der virtuelle Praktikumstag zur Nachwuchsgewinnung durchaus beitragen kann: So ziehen 35,8 % der Teilnehmer eine Facharztweiterbildung in der Urologie zumindest in Betracht.

Der virtuelle Praktikumstag wurde sehr gut angenommen und in kürzester Zeit von allen Studierenden erfolgreich absolviert, was durch die Beantwortung von MC-Fragen basierend auf den Inhalten der Videos als formale Anwesenheitskontrolle ermöglicht wurde. Es wird sich zeigen, wie die zukünftige praktische Ausbildung gestaltet werden kann und muss:

Die Vorteile virtueller Lehre liegen insbesondere darin, dass wichtige Lehrinhalte trotz z. B. einer Pandemie kontaktlos, sowie zeitlich und räumlich flexibel vermittelt werden können. Eindeutige Nachteile sind hingegen das nicht selbständige Ausführen und somit begrenzte Erlernen praktischer Tätigkeiten durch die Studierenden und die fehlende wichtige Interaktion zwischen den Studierenden und Dozenten [5]. Da es jedoch bei Praktikumstagen aber in erster Linie darum geht, dass die Studierenden essenzielle praktische Fertigkeiten selbständig in Interaktion mit Dozenten und anderen Studierenden üben bzw. erlernen, sollte ein virtuelles Format als Ersatz hier nicht dauerhaft zum Einsatz kommen, sondern zeitlich begrenzt Anwendung finden und eine Ergänzung (z. B. Vor‑/Nachbereitung) zum ursprünglichen praktischen Unterricht darstellen.

Der virtuelle Praktikumstag wird auch im kommenden Wintersemester 2020/2021 zum Einsatz kommen, da eine praktische Lehre im Rahmen eines von der Universität geplanten Hybridsemesters (Online-Vorlesungen, praktischer Unterricht in Anwesenheit unter Beachtung der Hygienevorgaben) nicht für alle Studierenden angeboten werden kann. Die vorhandenen Personalressourcen sind nicht ausreichend, um den Unterricht in dem Infektionsschutz angemessenen Minigruppen für alle Studierenden durchzuführen. Für urologisch Interessierte wird allerdings ein freiwilliger, aber reduzierter Präsenzpraktikumstag an 2 Tagen pro Monat mit einer Gruppengröße von maximal 5 Studierenden unter Einhaltung der Hygienevorschriften angeboten werden.

Zusammenfassend hat sich eine beeindruckende Beschleunigung der Digitalisierung in der medizinischen Lehre durch die COVID-19-Pandemie in nahezu allen medizinischen Fächern unserer Fakultät entwickelt. Es zeigt sich auch, dass die medizinische Lehre generell flexibel, aber auch mit Einschränkungen auf die COVID-19-Situation reagieren konnte und zukünftig vielleicht auch wieder bei ähnlichen Vorkommnissen reagieren muss. Ein Blick in die Vergangenheit zeigt, dass bereits im Rahmen der SARS-Pandemie 2002/2003 oder des MERS-Ausbruch 2015 in Südkorea aus den gleichen Gründen wie zu Zeiten von COVID-19 Ersatzangebote für die medizinische Präsenzlehre geschaffen werden mussten, die bereits damals teilweise in virtueller Form umgesetzt wurden [2, 4, 9].

## Fazit für die Praxis

- Mit unserem pragmatischen Vorgehen konnten wir mit Hilfe von studentischen Hilfskräften eine gute virtuelle Alternative für den urologischen Praktikumstag auf die Beine stellen.
- Dabei haben wir nach einem zuvor ausgearbeiteten Konzept viele kleine virtuelle Unterrichtseinheiten geschaffen.
- Durch die Verwendung unkomplizierter Tools und Lehrmittel war es möglich, in kurzer Zeit ein kontaktloses Lehrformat anbieten.
